# Post-Operative Benefits of Animal-Assisted Therapy in Pediatric Surgery: A Randomised Study

**DOI:** 10.1371/journal.pone.0125813

**Published:** 2015-06-03

**Authors:** Valeria Calcaterra, Pierangelo Veggiotti, Clara Palestrini, Valentina De Giorgis, Roberto Raschetti, Massimiliano Tumminelli, Simonetta Mencherini, Francesca Papotti, Catherine Klersy, Riccardo Albertini, Selene Ostuni, Gloria Pelizzo

**Affiliations:** 1 Department of the Mother and Child Health, Pediatric Unit, Fondazione IRCCS Policlinico San Matteo, Pavia, Italy; 2 Department of Internal Medicine, University of Pavia, Pavia, Italy; 3 Department of Child Neurology and Psychiatry C. Mondino National Neurological Institute, Pavia, Italy; 4 Brain and Behaviour Department, University of Pavia, Pavia, Italy; 5 Dipartimento di Scienze Veterinarie e Sanità Pubblica, Università degli Studi di Milano, Milano, Italy; 6 Department of Anaesthesiology and Intensive Care, Fondazione IRCCS Policlinico San Matteo, Pavia, Italy; 7 Biometry & Clinical Epidemiology, Scientific Direction, Fondazione IRCCS Policlinico San Matteo, Pavia, Italy; 8 Laboratory of Clinical Chemistry, Fondazione IRCCS Policlinico San Matteo, Pavia, Italy; 9 Pediatric Surgery Unit, Department of the Mother and Child Health, Fondazione IRCCS Policlinico San Matteo, Pavia, Italy; 10 Department of Clinical-Surgical, Diagnostic and Pediatric Sciences University of Pavia, Pavia, Italy; Eberhard Karls University, GERMANY

## Abstract

**Background:**

Interest in animal-assisted therapy has been fuelled by studies supporting the many health benefits. The purpose of this study was to better understand the impact of an animal-assisted therapy program on children response to stress and pain in the immediate post-surgical period.

**Patients and Methods:**

Forty children (3–17 years) were enrolled in the randomised open-label, controlled, pilot study. Patients were randomly assigned to the animal-assisted therapy-group (n = 20, who underwent a 20 min session with an animal-assisted therapy dog, after surgery) or the standard-group (n = 20, standard postoperative care). The study variables were determined in each patient, independently of the assigned group, by a researcher unblinded to the patient’s group. The outcomes of the study were to define the neurological, cardiovascular and endocrinological impact of animal-assisted therapy in response to stress and pain. Electroencephalogram activity, heart rate, blood pressure, oxygen saturation, cerebral prefrontal oxygenation, salivary cortisol levels and the faces pain scale were considered as outcome measures.

**Results:**

After entrance of the dog faster electroencephalogram diffuse beta-activity (> 14 Hz) was reported in all children of the animal-assisted therapy group; in the standard-group no beta-activity was recorded (100% vs 0%, p<0.001). During observation, some differences in the time profile between groups were observed for heart rate (test for interaction p = 0.018), oxygen saturation (test for interaction p = 0.06) and cerebral oxygenation (test for interaction p = 0.09). Systolic and diastolic blood pressure were influenced by animal-assisted therapy, though a higher variability in diastolic pressure was observed. Salivary cortisol levels did not show different behaviours over time between groups (p=0.70). Lower pain perception was noted in the animal-assisted group in comparison with the standard-group (p = 0.01).

**Conclusion:**

Animal-assisted therapy facilitated rapid recovery in vigilance and activity after anaesthesia, modified pain perception and induced emotional prefrontal responses. An adaptative cardiovascular response was also present.

**Trial Registration:**

ClinicalTrials.gov NCT02284100

## Introduction

The relationship between human beings and animals, especially dogs, has existed for thousands of years. Historically, animals have held an important role in this relationship as they provide company, stimulus and motivation. Animals are excellent company, since they do not discriminate or segregate anyone, that is, they are free of prejudice [1]. In spite of the long-lasting presence of companion animals in human life, the idea that interaction with animals may exert a positive effect on human health is rather recent [2].

The American Veterinary Medical Association classifies therapeutic animal assisted interventions (AAI) into three categories: animal assisted activities (AAA) that utilize companion animals; animal assisted therapy (AAT) that utilizes therapy animals and service animal programs (SAP) that utilize service animals. AAT in particular, is a goal-directed intervention in which an animal that meets specific criteria is an integral part of the treatment process. AAT is technically defined as the use of trained animals by trained health professionals to facilitate specific, measurable goals for individual patients for whom there is documented progress [3].

Interest in AAT has been fueled by studies supporting the many health benefits. AAT has proven a useful adjunct in a variety of settings including mental health facilitaties [4–10], nursing homes [11,12] and hospitals [13,14] where most studies have been performed with adult patients with variable interventions, goals, patient characteristics and patient needs. In these studies, AAT resulted in significant reductions in anxiety, agitation and fear. In children, AAT dogs decreased distress during painful medical procedures, promoted calmness in children with post-traumatic stress disorders and increased attention and positive behaviors in children with pervasive developmental disorders [10, 15–19].

Surgical procedures and hospitalization can be stressful for both children and their parents and they are associated with pain, helplessness, fear and boredom [19]. AAT has been shown to facilitate a child’s ability to cope with hospitalization, but to date, no studies on AAT benefits in pediatric surgery have been reported.

Emotion regulation is the adaptive behavioural response to stress. Emotions promote biochemically-mediated neurologic responses to emotionally-based stimuli [20]. Neurophysiologic measurement based on electrophysiological techniques can detect a wide range of dynamics of the emotional state by directly accessing the fundamental structure in the brain from which an emotional state emerges [21,22]. Hormonal indicators, such as cortisol level, and autonomic measurements, for example heart rate, blood pressure, can objectively detect the physiological responses of the endocrinological and autonomic nervous systems related to stress [22,23].

The purpose of this study was to better understand the effects of an AAT program on neurological, cardiovascular and endocrinological responses to stress and pain in the immediate post-operative period in children undergoing surgical procedures.

## Materials and Methods

### Ethics Statement

The study was performed according to the Declaration of Helsinki. The ethics committee of the Fondazione IRCCS Policlinico S. Matteo and Department of Internal Medicine, University of Pavia, approved the study protocol on April 11, 2013.

Animal-assisted therapy was also approved, on April 11, 2013, by the ethics committee of the Fondazione IRCCS Policlinico S. Matteo. No invasive intervention or drug sperimentation on the dog was performed; therefore the application of D.lgs. 116/92, European Directives 86/609/EE for the protection of animals used in scientific and experimental studies and 2010-63UE was not required. The dog owner provided consent for its use in the study.

Participant were recruited between September 01, 2013 and April 01, 2014.

The study was registered at ClinicalTrials.gov (Identifier: NCT02284100) after enrolment was initiated, because animal assisted-therapy was considered a complementary treatment. The authors confirm that all ongoing and the related trial for this drug/intervention are registered.

### Study protocol

The study protocol and supporting CONSORT checklist are available as supporting information; see S1 CONSORT Checklist and S1 and S2 Protocol.

**Fig 1:**
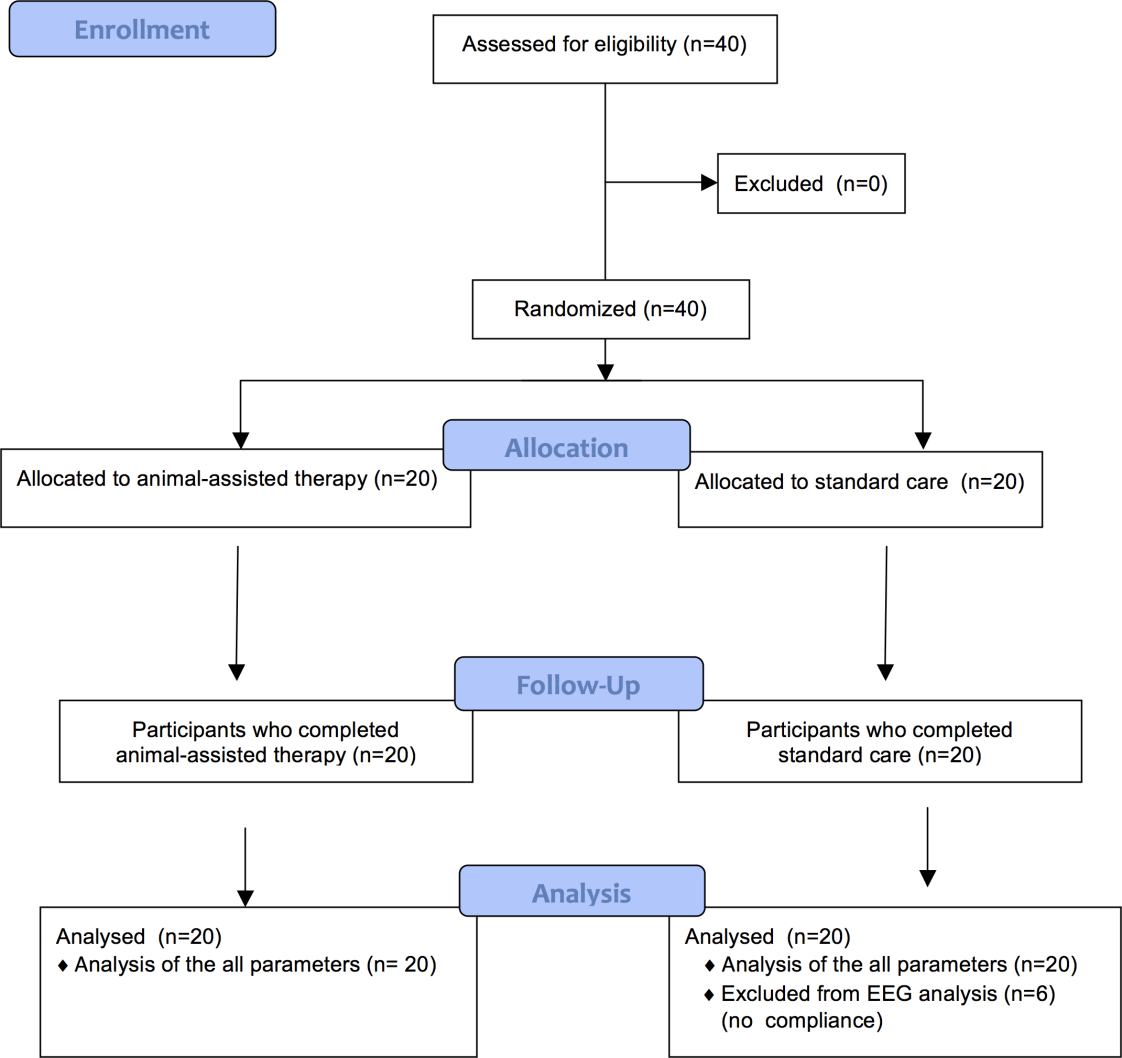
Flow diagram of the progress through the phases of the randomised trial of two groups (animal-assisted therapy and standard care after surgery).

This was a randomised open-label, controlled, pilot study. The different arms consisted of an experimental group with a AAT session after a surgical procedure and a control group with standard care after surgery. The flow diagram of the progress through the phases of randomization is reported in Fig 1.

The study variables were determined in each patient independently of the assigned group, before and after the experimental intervention, by a researcher unblinded to the patient’s group.

The outcomes of the study were to define the impact of AAT on neurological (primary endpoint), cardiovascular (secondary endpoint 2) and endocrinological signs (secondary endpoint 3), in response to stress and pain in children undergoing surgical procedures.

As outcome measures we considered:

- for neurological impact, the difference in the prevalence of beta (>14 Hz) electroencephalogram (EEG) activity between the intervention and control groups [24–27];
- for cardiovascular impact, the difference in vital signs (cerebral prefrontal oxygenation (HbO_2_) with near-infrared spectroscopy (NIRS), heart rate (HR), blood pressure (BP), oxygen saturation (SpO_2_) between intervention and control groups;
- for endocrinological impact, the difference in salivary cortisol levels, between the intervention and control groups.

The Wong-Baker Faces pain scale (FPS) was used to measure the child’s self-reported pain.

After trial commencement, no changes to the methods or trial outcomes were made.

### Participants

Forty immunocompetent children (both genders), aged 3 to 17 years, undergoing surgical procedures (including orchidopexy, inguinal or umbelical hernia repair, circumcision, varicocele treatment) were sequentially enrolled in this study.

A sample size of 20 patients per group for a total of 40 subjects was determined based dog availability. No data in the literature were available. We tentatively hypothesized a low prevalence of beta activity (primary endpoint) in the control group (with 2 different scenarios 5% and 10%), 80% power, alpha (2-tailed) = 0.10 (pilot study); the effect size (difference in prevalence between the 2 treatment groups) that could be elicited based on these assumptions was 31% and 34% respectively using a Chi2 test or 37% and 40% applying the continuity correction. The corresponding power obtainable using the Fisher exact test was 72% and 84% for the 2 scenarios, respectively Exclusion criteria included: allergy or fear of dogs, previous AAT experience, immunodeficiency, chronic illness, obesity and use of any medications.

In all subjects, surgery was performed between 8.30 am and 12 am and under general anaesthesia, at the Pediatric Surgery Unit, Fondazione IRCCS Policlinico S. Matteo, Pavia. Parental permission was obtained through a written and oral informed consent. Written assent by the patient was also obtained in children eight years of age and older before enrolment.

For the AAT session, a 7 year old Golden Retriever was employed as the therapy animal. Prior to the study, the dog underwent rigorous screening although she had previous experience in Animal Assisted Interventions and was already trained and prepared for this type of work. The dog was fully vaccinated, bathed regularly, screened for enteric pathogens, and treated for internal and external parasites on a monthly basis. The dog and handler met hospital policy for participating in animal-assisted therapy, including documentation of the dog’s current vaccinations, controllability and temperament.

The dog’s welfare was monitored by a veterinarian specialized specialist in “applied ethology and animal welfare”, European college of animal welfare and behavioural medicine (ECAWBM) certification.

### Follow-up

The participants were followed for the duration of the immediate postoperative period, an expected average of four hours.

### Procedures and data collection

At admission, auxological examination of the children included measurement of height, weight and body mass index. Height measurement was performed using a Harpenden stadiometer, performed with patients in an upright position, without shoes, with their heels together, arms extended down the sides of the body and head positioned parallel to the floor. Weight was measured with the children barefooted and wearing light clothes, standing upright in the centre of the scale platform with their arms extended down the sides of the body. BMI was calculated as body weight in kilograms divided by body height squared in meters.

Pre-surgery, the children were randomly assigned to the experimental AAT-group where the dog was present during post-operative awakening (2 hours after surgery) or the standard-group (STAND-group) where children had standard post-operative medical care. Randomization was carried out using opaque envelopes, half of which contained a paper that said “AAT-group” or “STANDARD-group” and was based on a permuted blocks randomization.

The AAT-group underwent a 20 min session with the AAT dog. The therapy dog was specially prepared and chosen for the interactions, which were evaluated as suitable and registered together with the handler. The handler monitored the dog, tended to the dog’s needs and answered questions about the dog.

The STAND-group underwent the same measurements over the same time frame, but without the dog being present.

EEG activity, cerebral prefrontal oxygenation, heart rate, blood pressure, oxygen saturation, salivary cortisol levels, faces pain scale were considered as indicators of neurological, cardiovascular and endocrinological response to stress and pain.

Data collection was performed in the following phases:

- for all parameters, post-operatively baseline (T1), two hours after surgery at re-admission to the Unit. Pre dog-intervention in the AAT-group;
- for all parameters, in the twenty minutes following T1 (T2). During the dog-intervention in the AAT-group. For the STAND-group, the child was asked to sit quietly;
- only for salivary cortisol levels, between 11 pm and midnight (T3, the time when cortisol is normally at its lowest)

### Electroencephalogram

Regarding the AAT-group patients, an EEG recording was obtained when the child was awake, 2 hours after surgery and before and during AAT intervention. In the STAND-group patients, and EEG recording was made 2 hours after surgery.

Any change in the normal physiological structure of the EEG, correlating the effect of the anaesthesia, post-operative stress of AAT intervention was recorded and monitored. In both cases, the protocol included an EEG recording tasting about 20 minutes, while awake, with open and closed eyes.

### Vitals signs

HR, BP, SpO_2_, HbO_2_ were monitored (Dräger Primus) and recorded as follows:

- T1 (every 5 minutes, for 10 minutes. Mean values were used for the statistical analysis);
- T2 (every 5 minutes, for 20 minutes. Mean values were used for the statistical analysis).

### Endocrinological parameters

Salivary cortisol levels were measured at T1, after T2 and T3. The saliva samples were collected using a standardized salivette and frozen at -20° until analysis in the laboratory. After thawing, salivary fluids were centrifugated to precipitate mucins and cortisol was assayed in the supernatant with a solid-phase radioimmunoassay, wherein 125-I labeled cortisol competes for a fixed time with cortisol in the biological sample (The Coat-A-Count Cortisol, Siemens, Los Angeles, CA).

### Pain response

The faces pain scale was used to measure the child’s self-reported pain at T1 and at the end of T2. This scale consisted of 6 cartoon faces with varying expressions ranging from very happy to very sad. The child rated the pain intensity on a scale, with point 0 being no pain and point 10 being the worst pain.

Analgesic treatment in the first 12 hours post-intervention was recorded.

### Anesthesia protocol

Written consent was obtained from the parents of the children scheduled for surgical procedures. All subjects were in good physical condition. Anesthesia included propofol (2–2.5 mh/g) as a sedative-hypnotic agent and fentanyl (1–1.5 mcg/kg) as the analgesic. After laryngeal mask or tracheal tube positioning, patients underwent volume controlled mechanical ventilation with an inspired mixture of air and oxygen using a closed breathing system (fresh gas flow of 0.75 l min^− 1^ oxygen and 1.5 l min^− 1^ air during anesthesia) adjusted to achieve an end-tidal carbon dioxide of 32–35 mmHg. Anesthesia was maintained via administration of Sevoflurane gas. Sevoflurane was administered in a 0.75 to 1.25 MAC range. Twenty minutes before the end of the intervention, all patients received Paracetamol 15 mg/kg, as an analgesic. At the end of the operation, the patient was transferred from the operating theater to the recovery room and subsequently was re-admitted to the Unit.

### Statistical analysis

Continuous variables were described as the mean and standard deviation (SD) or median and quartiles and categorical variables as counts and percentages. The prevalence of EEG beta activity was compared with the Fisher exact test. To test the effects of AAT on vital signs and on endocrinological parameters over time in the two groups, regression models for repeated measures were used, including a main effect for group and time, as well as their interaction; the latter was used to assess treatment effect (comparison of profiles over time). We also included a term for the duration of anesthesia, which was unbalanced between groups. Model assumptions were checked by visual inspection of the residual vs. fitted plot. Distribution of pain scores at the end of the session in the two groups was compared with the Fisher exact test. Differences between groups and 95% confidence intervals (95%CI) at each time point were computed. A 2-sided p-value<0.05 was considered statistically significant. No multiple test correction was applied given the exploratory nature of the pilot study. All statistical analyses were performed using Stata 13.1 (StataCorp, College Station, TX, USA).

## Results

The 40 children (32 M and 8 F; mean age 7.97±3.17 years; range 3.8–16.3), were randomly assigned to 1 of the 2 groups: the AAT-group (n = 20) or the STAND-group (n = 20), Fig 1.

Demographic, auxological features at admission and vital signs pre experimental intervention are reported in Table 1. The STAND-group showed a longer anesthesia time and lower cerebral oxygenation in comparison with the AAT-group; no correlation between these two variables was found (p = 0.5).

**Table 1:** Demographic and auxological features of the children at admission and vital signs pre intervention.

	Animal-assisted therapy group (n = 20)	Standard group (n = 20)
Age (yr)	8.59±3.70	7.36±2.48
Gender (M/F)	15/5	17/3
Height (cm)	131.00±23.59	120.70±17.83
Weight (kg)	31.50±17.18	25.35±10.38
BMI (kg/m^2^)	18.4±2.4	17.6±3.2
Anesthesia time (min)	45.0 (45.0–55.0) *	62.5 (50.0–70.0) *
Heart rate (bpm)	96.83±20.06	95.85±23.52
Systolic blood pressure (mmHg)	107.45±8.95	111.03±10.87
Diastolic blood pressure (mmHg)	65.55±7.37	66.20±7.99
Oxygen saturation (%)	98.65±1.04	98.47±0.94
Cerebral oxygenation-HbO_2_ (%)	79.5 (74.5–87.25) *	77.0 (67.5–79.00) *

*data are expressed as median (interquartile range)

### Primary endpoint

#### Electroencephalogram activity

A complete EEG recording was obtained in 20 patients in the AAT-group and 14 patients in the STAND-group (in 6 STAND-children, no compliance was obtained).

In 11/14 (80%) of the STAND-subjects and in 20/20 (100%) of the AAT-group patients the presence of atypical alpha-like (10–14 Hz) activity in anterior derivations (frontal and temporal) was observed during post-operative wakening (Fisher exact test p = 0.06).

In the STAND-group the alpha-like activity was unchanged with no beta activity recorded. On the contrary, after entrance of the dog faster EEG diffuse beta activity (> 14 Hz) was reported in all children of the AAT-group (0% vs 100%, p<0.001).

### Secondary endpoints

#### Vital signs data

Differences in treatment effect at every step during the intervention are detailed in Table 2. Given a marked inbalance in anesthesia time between groups, all models were adjusted for this difference.

**Table 2:** Vital signs measured in treated and standard groups. Differences and 95%CI are derived from repeated measurements models (including main effects for time and groups and their interaction) adjusted for anesthesia time.

Time	Heart rate (bpm)	Systolic blood pressure (mmHg)	Diastolic blood pressure (mmHg)	Oxygen saturation (%)	Cerebral oxygenation-HbO_2_ (%)
0	2.4 (-13.6 to 18.5)	-3.9 (-11.6 to 3.9)	-0.9 (-6.9 to 5.1)	-0.4 (-1.1 to 0.2)	7.1 (1.5 to 12.7)
1	7.4 (-4.0 to 18.9)	1.9 (-6.1 to 10.0)	-1.2 (-7.6 to 5.2)	2.0 (-3.4 to 7.5)	4.4 (-2.7 to 11.5)
5	6.6 (-2.9 to 16.0)	3.3 (-4.1 to 10.7)	-1.9 (-9.6 to 5.7)	2.6 (-2.9 to 8.1)	3.9 (-2.7 to 10.5)
10	15.7 (5.2 to 26.2)	1.5 (-6.0 to9.0)	3.5 (-4.4 to 11.4)	-0.3 (-1.0 to 0.5)	5.9 (0.3 to 11.4)
15	7.0 (-3.4 to 17.5)	2.1 (-6.1 to 10.3)	-2.1–8.5 to 4.2)	0.2 (-0.4 to 0.9)	5.0 (-1.0 to 10.9)
20	4.7 (-6.5 to 16.0)	2.4 (-5.9 to 10.8)	2.9 (-4.6 to 10.5)	-0.1 (-1.0 to 0.7)	6.6 (0.6 to 12.6)
**Treatment effect (p for interaction)**	**0.018**	**0.18**	**0.37**	**0.06**	**0.09**
*Change over time within STAND (p-value)*	*0*.*79*	*0*.*73*	*0*.*012*	*0*.*05*	*0*.*22*
*Change over time within AAT (p-value)*	*0*.*034*	*0*.*39*	*0*.*07*	*0*.*50*	*0*.*17*

**Fig 2:**
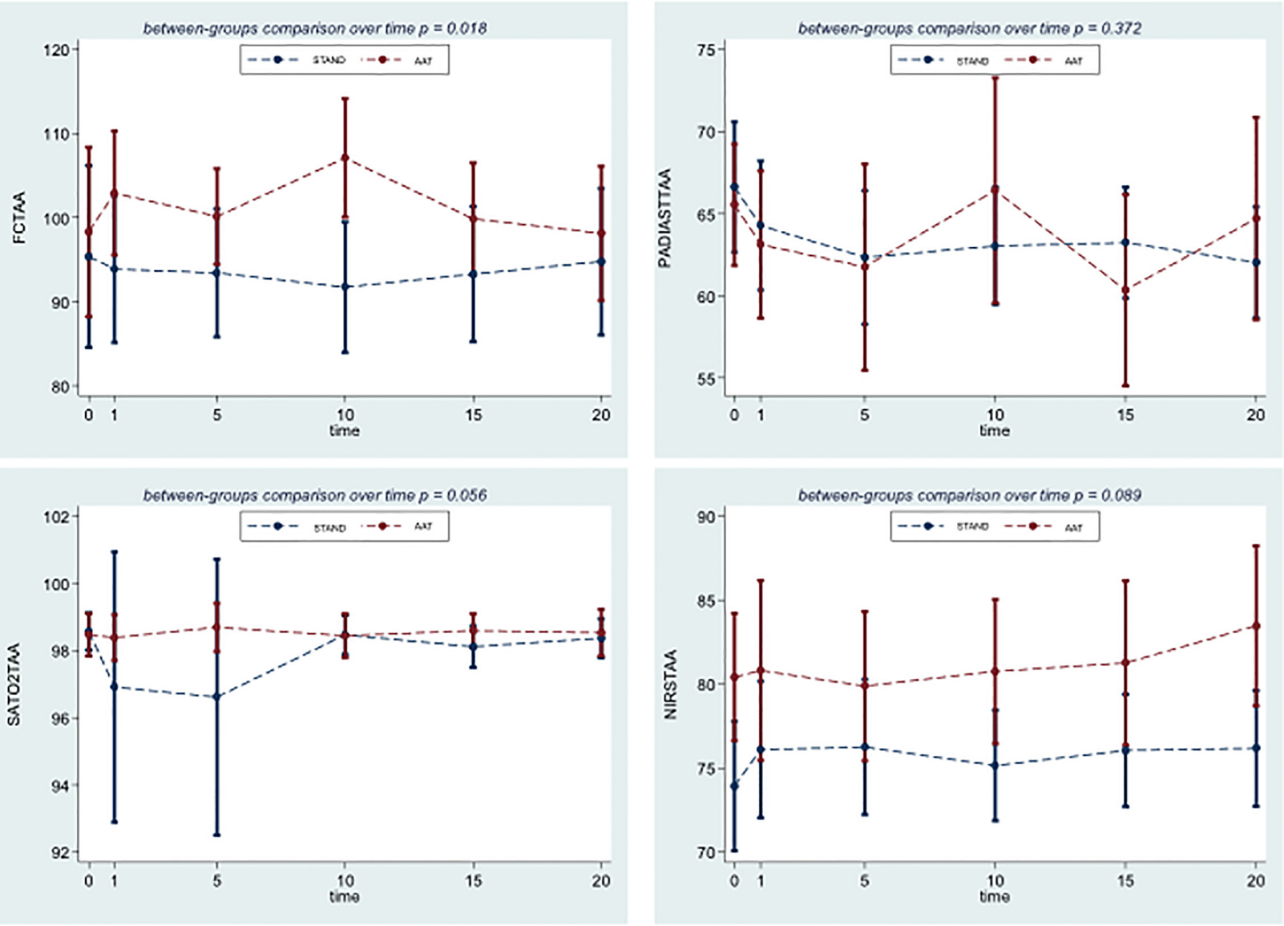
Comparison of the model-derived profiles for heart rate (HR), diastolic blood pressure (DBP), oxygen saturation (SpO_2_), cerebral prefrontal oxygenation (HbO_2_) in animal-assisted therapy (AAT) and standard (STAND) groups. All models are adjusted for time of anesthesia

Some differences in the time profile between groups were observed for heart rate, oxygen saturation and cerebral oxygenation. Only the former reached statistical significance (test for interaction p = 0.018), with heart rate increasing immediately after the start of observation in the AAT group, with the highest difference at 10 minutes, and decreasing afterwards. The difference between groups in oxygen saturation was highest at the beginning of the observation period, due to a decrease in the STAND group (test for interaction p = 0.06). Finally cerebral oxygenation tended to increase more in the AAT group in the later part of the observation period (test for interaction p = 0.09). Systolic and diastolic blood pressure were influenced by AAT, though a higher variability in DBP was observed. (Fig 2).

#### Endocrinological data

Repeated measurement of salivary cortisol levels did not show differences over time between the AAT and STAND-groups (test for interaction, p = 0.70). At T1, cortisol concentrations in the AAT and STAND groups were respectively 10.98±9.49 vs 14.17±18.2 nmol/L (Difference -3.2 nmol/L, 95%CI -13.4 to 7.0, p = 0.53), at T2, 14.94±18.09 vs 13.29±11.23 nmol/L (Difference 1.6, 95%CI -9.0 to 12.3, p = 0.75) and at T3, 7.89±9.71 vs 8.92±7.89 nmol/L (Difference -0.9, 95%CI -7.2 to 5.3, p = 0.76))

#### Pain scale findings

At T2, a lower perception of pain was noted in the AAT group in comparison with the STAND-group (p = 0.01), Table 3. Four cases in the AAT group had a reduction in pain from a level of six to zero.

**Table 3:** Faces pain scale in animal assisted therapy (AAT) and standard (STAND) groups (p = 0.01).

Pain levels	Number of patients (%)		
	AAT group (n = 20)	STAND group (n = 20)	Total
0	18 (66.67%)	9 (33.33%)	27 (100%)
2	2 (22.22%)	7 (77.78%)	9 (100%)
4	0 (0%)	3 (100%)	3 (100%)
6	0 (0%)	1 (100%)	1 (100%)

No differences in analgesic treatment in the first 12 hours post-intervention were recorded in the groups.

## Discussion

Surgery is one of the most stressful events a child may experience. The effects of AAT in pediatric surgery have not been described yet. We showed that AAT intervention during post-surgical procedures induces neurological and cardiovascular responses in the immediate post-operative period in children undergoing surgical procedures. Early vigilance recovery and activity after anaesthesia and activation of the emotional prefrontal area were noted in the AAT-group.

AAT has been utilized world-wide, as of 1960, and consists of animals with therapeutic training for patients with emotional, physical and mental diseases. In the presence of these animals, patient levels of anxiety and stress are reduced following painful procedures, physical activity is stimulated and interpersonal relationships, self-care and cardiovascular parameters are improved [19, 28–33].

The presence of atypical alpha-like (10–14 Hz) activity in anterior derivations (frontal and temporal) was observed during post-operative wakening in almost all of the STAND patients; this finding may be related to propofol treatment [34,35]. This activity, as reported in the literature, was constant throughout the duration of the postoperative recording, tending to fragment and disappear in the hours and days following anaesthesia. The 20 patients in the AAT-group, 2 hours after post-operative awakening, also showed the presence of atypical anterior alpha-like EEG activity. However, after the AAT intervention their EEG showed a quick and clear tendency to fragment the alpha-like activity up to its disappearance and replacement by quicker beta activity usually observed in awakening or during activities that require attention.

The more rapid disappearance of the propofol-induced atypical alpha-like anterior activity in patients in the AAT-group compared to the STAND-group is clear in our protocol; therefore, even though AAT stimulation is new and innovative (with few mentions in the literature), we can affirm that an early post-operative intervention with AAT stimulation could facilitate a rapid recovery of vigilance and activity after anaesthesia with propofol.

Hemodynamic changes during assessment of brain activity have been described [36]. The autonomic nervous system regulates involuntary body functions and is commonly evaluated by measuring systolic and diastolic blood pressure and heart rate reflex responses to physiological and pharmacological stimuli. In this study, the autonomic cardiovascular changes in HR and blood pressure could be considered as adaptative responses correlated to an early neurological response. Autonomic nervous system activity is an important component of human emotion. Mental processes influence bodily physiology, which in turn feeds back to influence thoughts and feelings. Afferent cardiovascular signals from arterial baroreceptors in the carotid sinuses are processed within the brain and contribute to this two-way communication with the body [22].

Several studies have investigated the pain-relieving effects of dog therapy in pediatric and adult patients and significant pain reduction has been reported reported [19,37]. EEG, although limited by its poor spatial resolution and high susceptibility to noise, has been widely used to investigate the brain dynamics relative to emotion as it enables the detection of immediate responses to emotional stimuli with excellent temporal resolution [21]. In the AAT-group the presence of beta activity was correlated with an increase in attention; these data could explain the finding of a higher threshold of pain sensitivity in these patients compared to the STAND-group, which instead had greater pain perception, presumably correlated to higher levels of drowsiness and apathy as demonstrated in the EEG findings.

The capacity to regulate emotions is particularly important during and after encountering a stressor. Emotional responses engage many different areas of the brain including parts of the limbic system and prefrontal cortex [38]. NIRS monitors hemodynamic activity in the brain measuring changes in oxygenated [HbO_2_] and deoxygenated haemoglobin [HHb] concentrations in regional cerebral blood flow. Exposure to emotionally-laden stimuli is known to produce measurable changes in chromophore concentration (ie [HbO_2_] and [HHb]). In this study, the prefrontal hemodynamic response as measured by NIRS, supports brain activity suggestive of central executive processing and a more positive activation of emotional areas during the AAT intervention.

In response to a stressor, the HPA axis (the major neuroendocrine system in mammals) regulates circulating levels of glucocorticoid hormones and provides rapid response and defense against stress. Cortisol is the main glucocorticoid, it is an essential hormone for normal homeostasis and is a useful indicator of short term stress in humans [23].

Brief dog therapy visits also result in a reduction in stress hormones [37]. Repeated measurement of salivary cortisol was employed to assess the endocrinological response on the HPA-axis during AAT. We used salivary cortisol because, unlike plasma, saliva collection is pain free for participants. With a delay of a few minutes, salivary cortisol represents an equivalent of the free, non-protein-bound cortisol in plasma [38]. A few studies have reported on the cortisol response to AAT. Barker et al measured serum cortisol in healthcare workers at baseline and one hour after a brief dog therapy visit or quiet rest and showed that a reduction in cortisol occurred with each intervention [4–7]. Viau et al [39] showed that the cortisol awakening response of children with autism is sensitive to the presence of service dogs, without any effect on the children's average diurnal cortisol levels. Beets et al reported benefits on stress modulation in male children with attachment issues [15,16]. We noted no different cortisol responses in the AAT and STAND-groups. The effect of AAT was probably not evident due to the high intra-individual biological variability of cortisol. However, the generally lower values found at T3 suggest a substantial integration of the HPA-axis after the surgical procedure. A possible explanation for the somewhat contradicting results might be the timing of the stressor, and according by different physiological stress systems might have an effect on emotion regulation [20]. To understand the real benefit of AAT on the stress response, an evaluation of other stress markers, such as epinephrine, norephinefrine, endorphin, oxitocin would be useful.

Notably, the use of AAT is not without risks [19] although no adverse outcomes resulted from this study. Without contact with oral secretions of the pets, the likelihood of transmission of infection from an immunized animal to an immunocompetent child is low [19]. Banks et al reported on 1690 patients visited by AAT dogs over a five year period and no zoonotic infections were reported [11,12]. AAT appears to be a therapeutic modality in which the benefits greatly outweigh the risks.

This pilot study was limited by the small sample size, thus further studies with a larger number of patients are mandatory to confirm the positive role of AAT after-surgery in children.

## Conclusion

Therapy dogs offer a novel and useful complementary therapy for children undergoing surgical procedures. AAT facilitates rapid recovery of vigilance and activity after anaesthesia, modifies pain perception and induces an emotional prefrontal response. An adaptative cardiovascular response was also observed.

## Supporting Information

S1 CONSORT Checklist CONSORT checklist. (PDF)

S1 Protocol Trial Protocol in Italian. (PDF)

S2 Protocol Trial protocol in English. (PDF)
