# Supplementary material for: Post-Operative Benefits of Animal-Assisted Therapy in Pediatric Surgery: A Randomised Study
Source: PLoS One. 2015 Jun 3;10(6):e0125813. doi: 10.1371/journal.pone.0125813 (PMC4454536; doi:10.1371/journal.pone.0125813)
Supplement: S1 Protocol — (PDF) [file pone.0125813.s002.pdf]

Your complimentary use period has ended. Thank you for using PDF Complete.
Click Here to upgrade to Unlimited Pages and Expanded Features

FONDAZIONE
[illegible]POLICLINICO ñSAN MATTEOò
[illegible]rattere Scientifico di diritto pubblico (D.M. 28/4/2006)
[illegible]3490189 - P. IVA 00580590180
[illegible] Golgi, 19 - 27100 PAVIA

PROTOCOLLO DI STUDIO

PROPONENTE	Prof.ssa Gloria Pelizzo
Servizio	UOC Chirurgia Pediatrica, Fondazione IRCCS Policlinico San Matteo e Università degli Studi di Pavia
Email	g.pelizzo@smatteo.pv.it
Telefono	0382-502910
Data	14/2/13
Titolo dello studio	**La risposta psiconeuroendocrina alla terapia complementare animale-assistita: modello di rilievo e controllo del dolore post operatorio**
1.Razionale	Il termine *Pet Therapy*, spesso impropriamente usato, fu coniato nel 1964 dal neuropsichiatra infantile Boris M. Levinson per descrivere lôuso di animali da compagnia nella cura di malattie psichiatriche. Levinson aveva infatti osservato come la presenza del proprio cane Jingles alle sedute con pazienti pediatrici con serie difficoltà di relazione e di comunicazione interpersonale, facilitasse lôinstaurarsi della relazione tra terapista e paziente, allôinizio di una terapia. Jingles sembrava avere la funzione di ñsciogliere il ghiaccioò aiutando il paziente ad abbassare le proprie barriere emotive. Secondo Levinson la chiave dellôefficacia terapeutica del partner animale sarebbe da ricercarsi nellôinstaurarsi di un rapporto empatico.<br>Lôutilizzo degli animali a fini terapeutici nel corso del tempo ha assunto unôimportanza crescente, supportato dalle positive evidenze scientifiche riportate in letteratura.<br>Il moderno termine di *Pet Therapy* si riferisce alla strutturazione metodologica dellôuso di soggetti animali finalizzata al trattamento di specifiche patologie. La *Pet Therapy* è stata riconosciuta come cura ufficiale dal Decreto del Presidente del Consiglio dei Ministri del 28 febbraio 2003. Tale Decreto ha sancito per la prima volta nella storia del nostro paese il ruolo che un animale può avere nella vita affettiva di una persona, nonché la valenza terapeutica degli animali da compagnia, finalizzata al trattamento di specifiche patologie.<br><br>Da un punto di vista operativo si distinguono due attività:<br>1) *Animal Assisted Activities* (AAA) ovvero ñAttività svolte con gli Animaliò, allo scopo di migliorare la qualità della vita di alcune categorie di persone (per esempio ciechi o portatori di handicap psico-fisici);<br>2) *Animal Assisted Therapies* o ñTerapie assistite con gli Animaliò (TAA). Lo scopo delle TAA consiste nellôaffiancare le terapie tradizionali utilizzando soggetti animali con specifiche caratteristiche. Le TAA possono essere utilizzate per migliorare lo stato fisico, sociale, emotivo e cognitivo di pazienti. Sono effettuate in ampi e differenti contesti e possono coinvolgere gruppi o singoli individui.<br><br>Attività e terapie assistite trovano un ambito di applicazione privilegiato soprattutto in ambito pediatrico.<br>Eôoramai dimostrato anche dai dati rilevati in letteratura che la presenza di un animale può:<br>- diminuire lo stress, lôansia, la paura, la noia e il dolore determinati dalle condizioni di salute e dalle situazioni derivanti dal ricovero (lontananza da familiari, dalla casa, dalle amicizie, dalle abitudini) in cui il bambino viene a trovarsi;<br>- facilitare e rendere più rapido il recupero dopo malattia;<br>- modificare il comportamento aumentando le capacità del bambino a partecipare al trattamento terapeutico, conducendolo al raggiungimento degli obiettivi prefissati. I bambini sono spesso estremamente fiduciosi e raggiungono facilmente un livello di intimità con lôanimale. Questo particolare legame fa dellôanimale un coterapeuta.

PDF Complete
Your complimentary use period has ended. Thank you for using PDF Complete.
Click Here to upgrade to Unlimited Pages and Expanded Features

	[illegible] nella sua diversità morfologica e comportamentale [illegible] il bambino nella formazione e nella ricchezza [illegible]io, offrendogli più modelli per i suoi processi elaborativi e [illegible] sua fantasia. Inoltre, lôinterazione con la diversità dellôanimale [illegible] fare riferimento alla diversità dellôanimale, aiuterebbe il [illegible]ôaffrontare la multiformità, trasformando la diffidenza in curiosità e tolleranza.<br>Il cane è lôanimale più largamente impiegato come co-terapeuta, sia nella cura di bambini che di adulti ed anziani. Mediante la sollecitazione al gioco e lôofferta di compagnia, stimola i pazienti allôinterazione.<br><br>Dai dati della letteratura si evince che:<br>1- lôAAT, applicata nei bambini per almeno 15 minuti, è un metodo efficace per ridurre il dolore di 4 volte;<br>2- stimoli emozionali sembrano sviluppare nel bambino risposte neuro endocrine ed immunitarie importanti (il contatto con lôanimale induce la liberazione di endorfine con conseguente stato di benessere, messa in circolo di linfociti che a loro volta incrementano la risposta immunitaria).<br><br>Nelle TAA lôattività svolta dal ñterapeuta animaleò nei confronti del ñpaziente uomoò è complessa e per il suo buon funzionamento richiede contributi provenienti da diverse discipline. Ogni TAA è il risultato di un lavoro sviluppato da un team interdisciplinare composto da numerose figure professionali che interagiscono sul campo, ciascuna con il proprio specifico ruolo ma in modo complementare.<br>I membri del gruppo di lavoro partecipano direttamente sia alla progettazione e alla valutazione dei programmi sia, in qualità di operatori, allo svolgimento della attività e delle terapie. Queste attività non devono mai risultare stressanti per lôanimale impiegato.
2 Obiettivo generale dello studio	Il ricovero in ospedale è da sempre motivo di paure e di tensioni sia per il bambino che per i familiari, di distacco dallôambiente domestico e dalle proprie abitudini, di somministrazione di terapie iniettive e di pratiche diagnostiche inevitabilmente male accettate, soprattutto in caso di ricovero per intervento chirurgico.<br>Il trauma psicologico conseguente si riverbera sia durante il ricovero, attraverso atteggiamenti di isolamento e di opposità alle cure dellôéquipe medica, sia al ritorno a casa, nei giorni immediatamente successivi alla dimissione, dove i bambini vivono difficoltà transitorie dôaddormentamento e di risvegli notturni, rifiutano il cibo, mostrano tutta una serie di paure mai dichiarate prima e manifestano episodi acuti di ansia da separazione dalle figure genitoriali.<br>Nellôambito di una terapie assistita con gli Animali, lôintroduzione di un aiuto-medico chirurgo inconsueto come il cane, può favorire la relazione tra bambino/famiglia-staff medico chirurgico, lôaccettazione delle cure, la diminuzione delle ansie e paure e il processo di guarigione.<br><br>Ci proponiamo pertanto di iniziare un progetto pilota di terapia assistita con cani nellôambito del percorso chirurgico del bambino, valutandone gli effetti positivi sul decorso post-operatorio.<br>Il miglioramento sarà valutato in termini di:<br>- Variazioni psiconeuroendocrine del periodo post-chirurgico, che possono influenzare la prognosi di guarigione con una migliore risposta allo stress e al dolore. In particolare saranno valutate:<br>a) la risposta neurofisiologica con monitoraggio dellôossigenazione tessutale cerebrale e somatica tramite Near-infrared spectroscopy (NIRS) e con registrazione EEG con una registrazione EEG in veglia e sonno e poligrafia. I dati neurofisiologici raccolti saranno poi elaborati con metodiche computerizzate atte a verificare eventuali modificazioni dei ritmi fondamentali che possano essere indicative di una risposta affettiva/ nocicettiva.<br>b) la risposta endocrinologica allo stress acuto, tramite il dosaggio della secrezione salivare di cortisolo.<br>c) la riduzione del periodo di terapia analgesia;<br>d) la percezione del dolore del bambino, tramite e somministrazione di test di valutazione scala del dolore (allegato 1)

PDF Complete
Your complimentary use period has ended. Thank you for using PDF Complete.
Click Here to upgrade to Unlimited Pages and Expanded Features

[illegible]	[illegible] dellâaccoglienza e assistenza per il bambino, tramite [illegible]one di questionari (allegato 2).
[illegible]	[illegible] variazione durante la registrazione dellâEEG della frequenza [illegible]a rispetto al basale
2.2 Obiettivi secondari ï (inclusi i sottostudi)<br>Come li misuro:	**End-point 2.1**<br>Confronto variazione HbO2 durante la registrazione NIRS,<br><br>**End-point 2.2**<br>Confronto variazione cortisolo salivare<br>-basale-dopo intervento TAA<br>-basale-cortisolo ore 23 (o anticipato, ma non prima delle 21)<br><br>**End-point 2.3**<br>Confronto numero di somministrazioni di analgesico richiesto nelle prime 12 ore dopo intervento e confronto della distribuzione delle risposte al test di valutazione della scala del dolore e del punteggio totale<br><br>**End-point 2.4**<br>Confronto della distribuzione delle risposte al questionario sullâaccoglienza e del punteggio totale
3 Piano di studio e disegno adottato	**X studio clinico controllato randomizzato (RCT) pragmatico**<br>**cecità: X no**; O singolo;<br>O doppio; O triplo<br>**disegno: X a gruppi paralleli**; O crossover;<br>O fattoriale; O altro é é é é é é é é<br><br>Randomizzazione a blocchi stratificata per età del bambino ≤6 anni e >6 anni. La randomizzazione avverrà al momento del prericovero. Dal punto di vista organizzativo i pazienti che accettano e vengono randomizzati al gruppo TAA saranno ricoverati nel giorno prefissato per la TAA, i bambini arruolati nel gruppo di controllo saranno ricoverati secondo la disponibilità della sala operatoria.<br>Prevista registrazione dello studio su sito pubblico di Clinical Trials<br><br>**Piano di studio**<br>**UTENTI**<br>Nei <u>bambini arruolati per la TAA</u> dopo 4 ore dallâintervento si procederà come segue:<br><br>1) Per 10 minuti prima dellâarrivo del cane (tracciato basale) e per 20 minuti in presenza dellâanimale, verranno eseguiti:<br>-monitoraggio dellâossigenazione tessutale cerebrale e somatica tramite Near-infrared spectroscopy (NIRS).<br>Tale metodica non invasiva fornisce la saturazione venosa regionale dellâossigeno dellâemoglobina (rSO2) del tessuto sotto il sensore; ovvero lâossigeno legato allâemoglobina rimanente dopo lâassorbimento da parte dei tessuti. Lâemoglobina ossigenata (HbO2) e lâemoglobina deossigenata (HHb) hanno spettri di assorbimento differenti nel NIR. Tale caratteristica permette di misurare separatamente le 2 forme di Hb e quindi la saturazione in ossigeno dellâHb (StO2) nei tessuti studiati.<br>I parametri ottenuti verranno utilizzati: 1) come indice di aumento del rischio ischemico e della compromissione della perfusione dei tessuti; 2) come indice indiretto di attivazione dellâarea pre-frontale da stimolazione nocicettiva ed emotiva. Infatti, è dimostrato che forti stimoli inducono cambi nella concentrazione di HbO2 e HHb per aumento del flusso sanguigno.<br>-registrazione EEG in veglia e in sonno (occhi chiusi) con registrazione dei parametri poligrafici di ECG, respirogramma e attività muscolare.<br>Sebbene in letteratura siano scarse le segnalazioni relative alla capacità dellâEEG di superficie a quantificare possibili variazioni neurofisiologiche correlate allo stress, verranno effettuate registrazioni poligrafiche che permettano di acquisire un tempo sufficiente di attività elettrica cerebrale sia in veglia che in sonno. I dati raccolti in modalità digitale, saranno elaborati

PDF Complete
Your complimentary use period has ended. Thank you for using PDF Complete.
Click Here to upgrade to Unlimited Pages and Expanded Features

[illegible] odiche computerizzate per cercare di dimostrare eventuali [illegible] ritmi fondamentali con studio di mappe e di spettri. In [illegible] anno studiate le possibili variazioni delle componenti beta e [illegible] cerebrali e, nel sonno, eventuali modificazioni della struttura [illegible] sonno. Lo studio poligrafico permetterà di valutare le [illegible] lei parametri di base e un loro possibile correlato allo stress .
Risposte emotive/nocicettive diverse corrispondono ad una diversa attivazione delle aree corticali valutabile con EEG e con NIRS (l’attivazione di un’area corticale porta ad un incremento del flusso cerebrale senza un proporzionale del consumo di ossigeno e conseguentemente ad un aumento dell’emoglobina ossigenata e decremento dell’emoglobina deossigenata).
- monitoraggio SpO2, frequenza cardiaca, frequenza respiratoria

2) Prima dell’arrivo del cane, al termine della sessione di TAA e alle 23 del giorno dell’intervento (nadir fisiologico di secrezione) verranno eseguito dosaggi del cortisolo salivare, come indicatore endocrinologico di stress.
Qualora le esigenze del piccolo lo ritengano necessario, per rispettare il ritmo sonno-veglia, il prelievo potrà essere anticipato (non prima delle ore 21).
Una migliore risposta allo stress induce un minor incremento della secrezione di cortisolo. La valutazione del cortisolo salivare è una metodica non invasiva la cui attendibilità è dimostrata da numerosi lavori scientifici anche recenti.

Nei bambini appartenenti al <u>gruppo di controllo</u> il monitoraggio NIRS e dei parametri vitali e la registrazione dell’EEG verranno eseguiti a 4 ore dall’intervento con registrazione di 30 minuti (i primi 10 minuti rappresenteranno il tracciato di controllo basale). Nei bambini appartenenti al <u>gruppo di controllo</u> i monitoraggi EEG e dei parametri vitali verranno eseguiti a 4 ore dall’intervento con registrazione di 30 minuti. L’esecuzione dell’EEG non prevede in alcun modo l’alterazione del ritmo sonno-veglia del bambino e il piccolo non verrà svegliato per la registrazione. Qualora nel momento dell’esecuzione dell’esame il bambino dormisse, verrà ritirato dallo studio. Il dosaggio del cortisolo salivare sarà eseguito dopo 4 ore e alle ore 23. Qualora le esigenze del piccolo lo ritengano necessario, per rispettare il ritmo sonno-veglia, il prelievo potrà essere anticipato (non prima delle ore 21).

I bambini del gruppo di controllo pur non beneficiando degli eventuali vantaggi forniti dalla presenza dell’animale, l’esecuzione del monitoraggio dell’attività elettrica cerebrale e dei parametri vitali offrirà il vantaggio di evidenziare precocemente complicanze asintomatiche secondarie all’anestesia.

<u>In entrambe i gruppi</u>

3)somministrazione al paziente di test di valutazione scala del dolore (6 faccette, vedi allegato 1) dopo 4 ore dall’intervento. La scala analogica visiva utilizzata è semplice e, come da linee guida, può essere utilizzata a partire dai 3 anni e in alcuni bambini anche prima

4) partire da 4 ore dopo l’intervento (periodo corrispondente al termine dell’effetto della terapia anestesiologica) in tutti i bambini verrà registrato il numero di somministrazioni di analgesico richiesto nelle 12 ore successive all’intervento

5) prima della dimissione verrà consegnato questionario di valutazione della qualità dell’accoglienza e assistenza (allegato 2)

ANIMALI
Durante le sessioni con l’animale verrà redatto un questionario (indice di valutazione dello stato di benessere dell’animale) che dovrà essere compilato dall’operatore (allegato 3).
In base al tipo di paziente verranno valutate le reazioni comportamentali allo

Your complimentary use period has ended. Thank you for using PDF Complete.

Click Here to upgrade to Unlimited Pages and Expanded Features

[illegible]viduare, in relazione allôetà (fase verbale e non verbale), [illegible]ado di disabilità del paziente, le reazioni dellôanimale.
[illegible]tività del cane vengono pertanto riportate:
[illegible] di intervento e utenza
[illegible]e tempi di impiego
[illegible]ero utenti con cui lôanimale ha avuto contatto

4 Soggetti

CRITERI DI ARRUOLAMENTO

BAMBINI

40 pazienti che afferiscono al Reparto di Chirurgia Pediatrica.

Lôinclusione nello studio avverrà con il consenso informato scritto dei genitori e lôassenso scritto del minore.

<u>*Criteri di arruolamento:*</u>

- età compresa tra 3-17 anni
- sesso maschile e femminile

pazienti sottoposti ad intervento di chirurgia minore addominale e plastica pediatrica ricostruttiva via *open* o laparoscopica (chirurgia dôurgenza differibile o di elezione), in cui a) non siano stati posizionati drenaggi; b) le cicatrici siano contenute (approccio mini invasivo o piccoli cicatrici con suture intradermiche); c) il decubito del bambino non sia obbligato; d) non vi siano accessi venosi centrali. In particolare erniorrafie, criptorchidismi, piccole chirurgie della parete addominale, exeresi di lesioni limitate alla fascia, circoncisioni, correzioni malformazioni cute e sottocute degli arti e del tronco, medicazioni di ustioni.

Per tali interventi la terapia analgesica post-operatoria prevede una somministrazione di paracetamolo per via endovenosa al termine dellôintervento e le successive somministrazioni ad intervalli di 4 ore al bisogno.

<u>Criteri di esclusione:</u>

-allergia al pelo di cane
-condizioni di immunodeficienza
-assunzione di terapia steroidea. In caso di pregressa terapia, questôultima deve essere stata sospesa da almeno 1 mese
-assunzione di farmaci in grado di interferire con la funzionalità surrenalica
-assunzione di farmaci anticomiziali
-fobia del cane

Saranno formati due gruppi secondo randomizzazione a blocchi stratificati per età:
-GRUPPO 1: 20 bambini che faranno il percorso post operatorio accompagnato dal cane
-GRUPPO 2: 20 bambini arruolati che faranno il normale percorso post-operatorio senza ausilio dellôanimale

I bambini arruolati per le TAA saranno ricoverati in stanza singola dedicata.

REQUISITI ANIMALE IMPIEGATO PER TAA

Per le attività di terapia complementare animale-assistita, verrà impiegato un cane di razza *golden retriver* con i seguenti requisiti:
-Il cane è in possesso di idoneità sanitaria ed è periodicamente sottoposto a controlli veterinari con aggiornamento calendario vaccinale previsto
-Il cane è in possesso di idoneità comportamentale, è docile e risponde correttamente ai comandi del suo istruttore
-Semestralmente il cane viene sottoposto a esami delle feci, trattamento anti-parassitario
-Il cane non presenta perdita eccessiva di pelo, né malattie dermatologiche e/o orali
-Il cane viene trasportato in un mezzo igienicamente idoneo. La distanza tra mezzo di trasporto e reparto segue il percorso più breve (vedi allegato 4)
-Lôigiene del cane nelle 24 ore prima della TAA prevede bagno, taglio e

Your complimentary use period has ended. Thank you for using PDF Complete.
Click Here to upgrade to Unlimited Pages and Expanded Features

	[illegible]ghie, pulizia degli occhi e delle orecchie, spazzolamento con [illegible] superflui<br><br>[illegible] presenza di accessi venosi, flebo o parti immobilizzate, [illegible] posizionato nella parte opposta<br><br>Gli operatori che vengono a contatto con lôanimale devono provvedere alla pulizia e disinfezione delle mani con alcol gel o sapone, prima di ogni contatto con il bimbo.<br><br>Lo stato di salute viene dichiarato ad ogni seduta dal Coadiutore dellôanimale e la valutazione clinica ogni 6 mesi da un Medico Veterinario.<br>Verrà garantito il benessere psico-fisico dellôanimale durante le attività/terapie tramite compilazione di schede di osservazione comportamentale (vedi allegato 3).<br>Durante lôarrivo e la permanenza dellôanimale nella struttura, verranno rispettate le norme igieniche necessarie per non creare disagi sanitari in un ambiente chirurgico.<br>Il cane avrà accesso ad una sola stanza di degenza adibita a tal fine ed isolata dal resto del reparto. Il cane ñopereràò adagiato su un supporto posizionato accanto al letto, ad una altezza compatibile con il contatto con il bambino.<br>Il cane, accompagnato dall'addestratore, arriverà presso la nostra unità operativa in giorni e orari concordati. All'ingresso del reparto si provvederà alla pulizia delle zampe del cane con salviettine disinfettanti (clorexidina su base acquosa). Il cane verrà accompagnato dal bambino seguendo un percorso prestabilito fisso, che terrà conto del tragitto più breve per arrivare alla stanza di degenza (vedi allegato 4). Il tempo di permanenza in stanza per ogni seduta è di 30 minuti. Allôuscita del cane si provvederà alla pulizia e disinfezione della stanza e al cambio lenzuola. Lôuscita del cane seguirà la stessa via utilizzata per lôingresso (vedi allegato 4)
5 Trattamenti	Terapia animale assistita
5.1 Piano per il trattamento	Terapia animale assistita *vs standard of care*
5.2 Randomizzazione	Verrà preparata una lista di randomizzazione a blocchi stratificata per età del bambino ≤ 6 anni e >6 anni dal Servizio di Biometria
6 Durata dello studio e Valutazioni previste	Durata 1 anno<br>Tutte le valutazioni vengono eseguite solo nel periodo post-operatorio
7.1 Elementi per il calcolo della numerosità del campione o della potenza<br>7.2 Calcolo numerosità del campione/potenza	Il calcolo della numerosità campionaria è basato sulle seguenti ipotesi α (2 code)=0.10 (studio pilota), potenza 80%. Con 20 pazienti per gruppo sarà possibile mettere in evidenza un ñ*effect size*ò pari a 0.8 deviazioni standard. La numerosità campionaria è stata calcolata utilizzando lôend point primario
7.3 Piano di analisi obiettivo primario	**End-point 1**<br>Confrontare la variazione durante la registrazione dellôEEG della frequenza delle onda beta rispetto al basale<br><u>Nel gruppo TAA</u><br>Per 10 minuti prima dellôarrivo (tracciato basale) del cane e per 20 minuti in presenza dellôanimale verrà eseguita registrazione EEG.<br><u>Nel gruppo di controllo</u><br>Registrazione EEG a 4 ore dallôintervento con registrazione di 30 minuti (i primi 10 minuti rappresenteranno il tracciato basale)<br><br><u>Test statistico</u>: Confronto della differenza di frequenza delle onde beta mediante test t di Student o test di U Mann-Whitney, Calcolo della differenza media tra gruppi e relativo intervallo di confidenza al 95%.
7.4 Piano di analisi obiettivi secondari	**End-point 2.1**<br>Confronto variazione HbO2 durante la registrazione NIRS<br><u>Nel gruppo TAA</u><br>Per 10 minuti prima dellôarrivo (tracciato basale) del cane e per 20 minuti in presenza dellôanimale verrà eseguito monitoraggio NIRS<br><u>Nel gruppo di controllo</u><br>Monitoraggio NIRS a 4 ore dallôintervento con registrazione di 30 minuti (i primi 10 minuti rappresenteranno il tracciato basale)

Your complimentary use period has ended. Thank you for using PDF Complete.
PDF Complete
Click Here to upgrade to Unlimited Pages and Expanded Features

	[illegible]: Confronto mediante test t di Student o test di U Mann-[illegible]olo della differenza media tra gruppi e relativo intervallo di [illegible]95%.<br><br>Confronto variazione cortisolo salivare<br>Nel gruppo TAA<br>Prima dell'arrivo del cane, al termine della sessione di TAA e alle 23 del giorno dell'intervento (nadir fisiologico di secrezione) verrà eseguito dosaggio del cortisolo salivare<br>Nel gruppo di controllo<br>Il dosaggio del cortisolo salivare sarà eseguito dopo 4 ore e alle ore 23.<br><br>Qualora le esigenze del piccolo lo ritengano necessario, per rispettare il ritmo sonno-veglia, il prelievo per il cortisolo salivare delle ore 23 potrà essere anticipato (non prima delle ore 21).<br><br>Test statistico: Confronto mediante test t di Student o test di U Mann-Whitney. Calcolo della differenza media tra gruppi e relativo intervallo di confidenza al 95%.<br><br>**End-point 2.3**<br>Confronto numero di somministrazioni di analgesico richiesto nelle prime 12 ore dopo intervento e confronto della distribuzione delle risposte al test di valutazione della scala del dolore<br>Nei due gruppi verrà somministrato test di valutazione scala del dolore (6 faccette, vedi allegato 1) dopo 4 ore dall'intervento e a partire da 4 ore dopo l'intervento verrà registrato il numero di somministrazioni di analgesico richiesto nelle 12 ore successive all'intervento<br>Test di Fisher per il confronto della terapia analgesica e della distribuzione delle risposte<br>Test di U Mann-Whitney per il confronto dei punteggi<br><br>**End-point 2.4**<br>Confronto della distribuzione delle risposte al questionario sull'accoglienza e del punteggio totale<br>Al momento della dimissione verrà consegnato questionario di valutazione della qualità dell'accoglienza e assistenza (allegato 2)<br><br>Test di Fisher per il confronto della distribuzione delle risposte<br>Test di U Mann-Whitney per il confronto dei punteggi
7.5 CRF e gestione dati	Vedi allegato (tabella excell)
8 Consenso informato	Secondo normativa (vedi consensi allegati)
9 Costi	Non sono previsti costi a carico della Fondazione. Per la spesa degli accertamenti extra-routine saranno ricoperte con ricavati da *fund raising*
10 Personale coinvolto e ruolo	-Prof.ssa Gloria Pelizzo, UC Chirurgia Pediatrica, Fond. IRCCS Policlinico S. Matteo Pavia e Università degli Studi di Pavia. Ruolo: chirurgo<br>-Prof.ssa Daniela Larizza, Dott.ssa Valeria Calcaterra, US Endocrinologia e Diabetologia Pediatrica, Fond. IRCCS Policlinico S. Matteo e Università degli Studi di Pavia. Ruolo: valutazione endocrinologica<br>-Prof. Antonio Braschi, Dr.ssa Simonetta Mencherini, UC Anestesia e Rianimazione 1, Fond. IRCCS Policlinico San Matteo Pavia. Ruolo: anestesista<br>-Prof. Pierangelo Veggiotti, US epilettologia dell'infanzia e dell'adolescenza, SC Neuropsichiatria Infantile, Fond. IRCCS Istituto Neurologico Nazionale Casimiro Mondino. Ruolo: valutazione neuropsichiatrica<br>-Dott. Annalisa Marchetti, Servizio Veterinario, Direzione Scientifica, Fond. IRCCS Policlinico S. Matteo Pavia. Ruolo: valutazione veterinaria<br>-Dott.ssa Clara Palestrini, Dipartimento di Scienze Veterinarie e Sanità Pubblica, Facoltà di Medicina Veterinaria, Università degli Studi di Milano. Ruolo valutazione veterinaria-comportamentalista<br>-Dott.ssa Debra Buttram, Natura Animale, Istruttore Cani Servizio e Hearing Dogs-Membro Taskforce di Animal Assisted Interactions International. Ruolo: coadiutore cane

Your complimentary use period has ended. Thank you for using PDF Complete.
Click Here to upgrade to Unlimited Pages and Expanded Features

[illegible]	[illegible]erine Klersy, Servizio di Biometria ed Epidemiologia Clinica, [illegible]entifica, Fondazione IRCCS Policlinico San Matteo). *Ruolo:* [illegible]tatistica dei dati
[illegible]	[illegible]
[illegible] pubblicazioni	[illegible]ntatori

Firma del proponente
Prof.ssa Gloria Pelizzo

PDF Complete
Your complimentary use period has ended. Thank you for using PDF Complete.
Click Here to upgrade to Unlimited Pages and Expanded Features

[illegible]an brain EEG indices of emotions: delineating responses to affective vocalizations by measuring frontal theta event-related synchronization.Neurosci Biobehav Rev. 2011 Oct;35(9):1959-70.

Braun C, Stangler T, Narveson J, Pettingell S. Animal-assisted therapy as a pain relief intervention for children. Complement Ther Clin Pract. 2009;15:105-109.

Chakravarti S, Srivastava S, Mittnacht AJC. Near Infrared Spectroscopy (NIRS) in Children. Seminars in Cardiothoracic and Vascular Anesthesia 2008; 170-78.

Cirulli F, Borgi M, Berry A, Francia N, Alleva E. Animal-assisted interventions as innovative tools for mental health. Ann Ist Super Sanita. 2011;47:341-348.

Coakley AB, Mahoney EK. Creating a therapeutic and healing environment with a pet therapy program. Complement Ther Clin Pract. 2009;15:141-146.

Damasio AR, Grabowski TJ, Bechara A, Damasio H, Ponto LL, Parvizi J, Hichwa RD.Subcortical and cortical brain activity during the feeling of self-generated emotions. Nat. Neurosci., 3 (2000), pp. 1049ï 1056

Dean Kurth C and Brian Uher BS. Cerebral Hemoglobin and Optical Pathlength Influence Near-Infrared Spectroscopy Measurement of Cerebral Oxygen Saturation. Anesth Analg 1997;84:1297-1305.

Dimitrijeviĺ I. Animal-assisted therapy-a new trend in the treatment of children and adults. Psychiatr Danub. 2009;21:236-241.

Fitzgerald FT. The therapeutic value of pets. West J Med. 1986 Jan;144(1):103-105.

Grandgeorge M, Hausberger M. Human-animal relationships: from daily life to animal-assisted therapies. Ann Ist Super Sanita. 2011;47:397-408.

Harmon-Jones E, Winkielman P (Eds.), Asymmetrical frontal cortical activity, affective valence, and motivational direction. Social Neuroscience, Guilford Press, New York (2007), pp. 137ï 156

Herrmann MJ, Ehlis AC, Fallgatter AJ. Prefrontal activation through task requirements of emotional induction measured with NIRS. Biol Psychol. 2003;64:255-263.

Hogue CM, Fry MD, Fry AC, Pressman SD. The influence of a motivational climate intervention on participants' salivary cortisol and psychological responses. J Sport Exerc Psychol. 2013 Feb;35(1):85-97.

Jausovec N, Jausovec K, Gerlic I. Differences in event-related and induced EEG patterns in the theta and alpha frequency bands related to human emotional intelligence. Neurosci. Lett., 311 (2001), pp. 93ï 96

Jofré M L. Animal- assisted therapy in health care facilities. Rev Chilena Infectol. 2005;22:257-63.

King C, Watters J, Mungre S. Effect of a time-out session with working animal-assisted therapy dogs. Journal of Veterinary Behavior: Clinical Applications and Research, Volume 6, Issue 4, Julyï August 2011, Pages 232-238.

Lloyd-Fox S, Blasi A, Elwell CE. Illuminating the developing brain: The past, present and future of functional near infrared spectroscopy Neuroscience and Biobehavioral Reviews 2010;34 :269ï 284.

Looser RR, Metzenthin P, Helfricht S, Kudielka BM, Loerbroks A, Thayer JF, Fischer JE. Cortisol is significantly correlated with cardiovascular responses during high levels of stress in critical care personnel. Psychosom Med. 2010;72:281-289.

PDF Complete
Your complimentary use period has ended. Thank you for using PDF Complete.
Click Here to upgrade to Unlimited Pages and Expanded Features

[illegible]ster RP, Marin MF, Francois N, Sindi S, Wan N, Findlay H, [illegible] J, Corbo V, Dedovic K, Lai B, Plusquellec P. The DeStress for [illegible] Education Program on Cortisol Levels and Depressive [illegible]e Transition to High School. Neuroscience. 2013 Feb 8. pii: [illegible]oscience.2013.01.057

Lust E, Ryan-Haddad A, Coover K, Snell J. Measuring clinical outcomes of animal-assisted therapy: impact on resident medication usage. Consult Pharm. 2007;22:580-585.

Marcus DA, Bernstein CD, Constantin JM, Kunkel FA, Breuer P, Hanlon RB. Animal-assisted therapy at an outpatient pain management clinic. Pain Med. 2012;13:45-57.

Michels N, Sioen I, Braet C, Huybrechts I, Vanaelst B, Wolters M, De Henauw S. Relation between salivary cortisol as stress biomarker and dietary pattern in children. Psychoneuroendocrinology. 2013 Jan 15. pii: S0306-4530(12)00454-4. doi: 10.1016/j.psyneuen.2012.12.020. [Epub ahead of print]

Miller J, Ingram L. Perioperative nursing and animal-assisted therapy. AORN J. 2000;72:477-483.

Muñoz Lasa S, Ferriero G, Brigatti E, Valero R, Franchignoni F. Animal-assisted interventions in internal and rehabilitation medicine: a review of the recent literature. Panminerva Med. 2011;53:129-136.

Nagamitsu S, Yamashita Y, Tanaka H,Toyojiro Matsuishi T. Functional near-infrared spectroscopy studies in children. BioPsychoSocial Medicine 2012, 6:7.

Piva E, Liverani V, Accorsi PA, Sarli G, Gandini G. Welfare in a shelter dog rehomed with Alzheimer patients. Journal of Veterinary Behavior: Clinical Applications and Research, Volume 3, Issue 2, Marchï April 2008, Pages 87-94.

Ranabir S, Reetu K. Stress and hormones. Indian J Endocrinol Metab. 2011;15:18-22.

Reed R, Ferrer L, Villegas N. Natural healers: a review of animal assisted therapy and activities as complementary treatment for chronic conditions. Rev Lat Am Enfermagem. 2012;20:612-618.

Tai K, Chau T. Single-trial classification of NIRS signals during emotional induction tasks: towards a corporeal machine interface. J Neuroeng Rehabil. 2009;6:39.

Viau R, Arsenault-Lapierre G, Fecteau S, Champagne N, Walker CD, Lupien S. Effect of service dogs on salivary cortisol secretion in autistic children. Psychoneuroendocrinology. 2010;35:1187-1193.

Yang H, Zhou Z, Liu Y, Ruan Z, Gong H, Luo Q, Lu Z. Gender difference in hemodynamic responses of prefrontal area to emotional stress by near-infrared spectroscopy. Behav Brain Res. 2007;178:172-176.

Your complimentary use period has ended. Thank you for using PDF Complete.
Click Here to upgrade to Unlimited Pages and Expanded Features

ALLEGATO 1

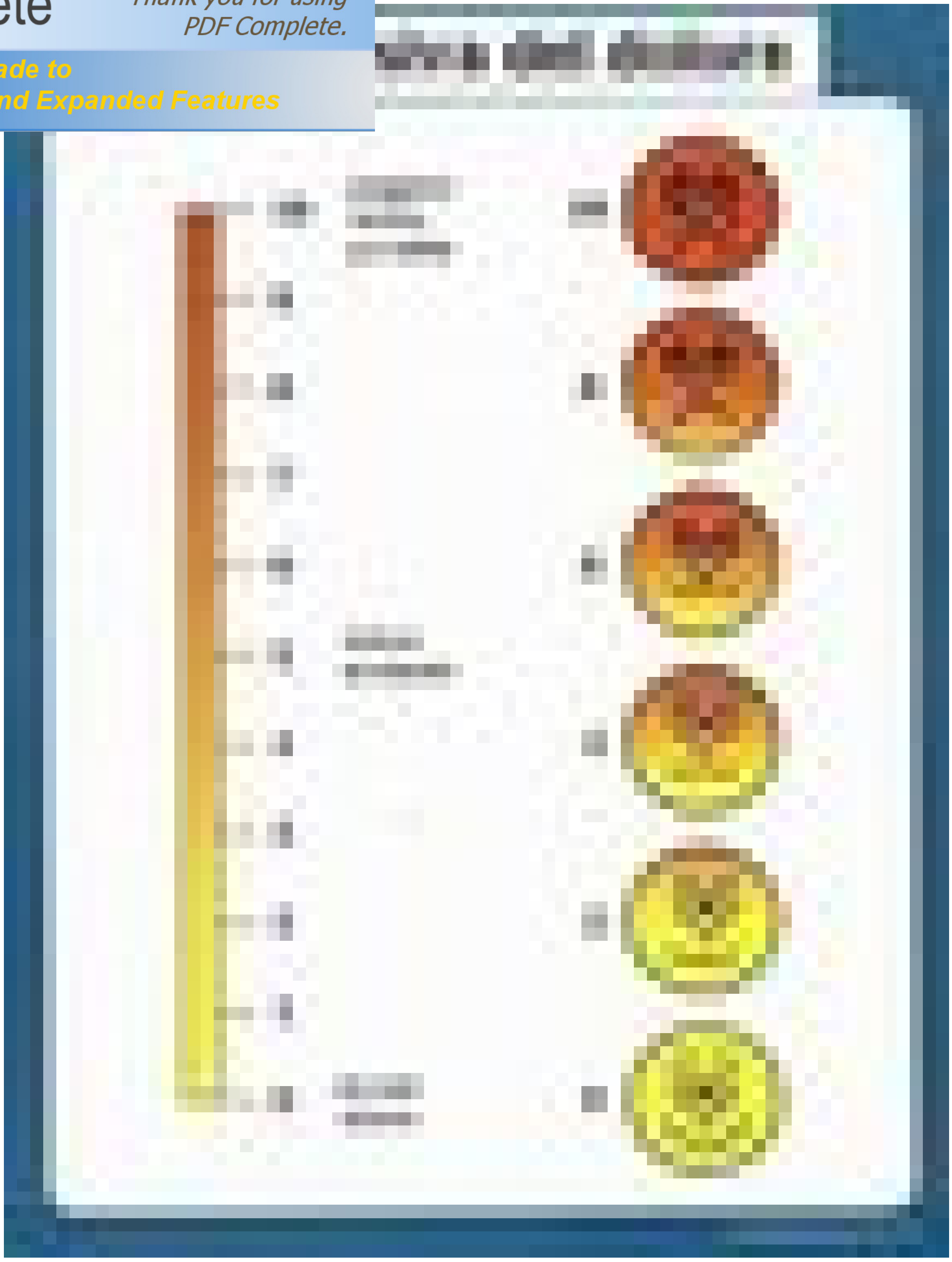

Your complimentary use period has ended. Thank you for using PDF Complete.
Click Here to upgrade to Unlimited Pages and Expanded Features

☐ Madre
☐ Padre
☐ Nonno/nonna
☐ Altro famigliare

Regione in cui risiede la famiglia del minore portato in Ospedale________________

Provincia in cui risiede la famiglia del minore portato in Ospedale________________

Sesso del bambino
☐ Femmina
☐ Maschio

Età del bambino in anni all'atto del ricovero
☐ Da 1 a 4
☐ Da 5 a 9
☐ Da 10 a 14
☐ Da 15 a 17

Tipo di intervento a cui verrà sottoposto il bambino_______________________________________

I) Come giudica l'accoglienza in reparto?
1 Pessima
2 Scarsa
3 Sufficiente
4 Buona
5 Ottima

II) Quanto è dôaccordo con la seguente affermazione: la durata del ricoevro è eccessiva:
1 Per nulla
2 Abbastanza
3 Dôaccordo
4 Molto dôaccordo
5 Completamente dôaccordo

III) Come giudica il confort della stanza del reparto dove il bimbo è stato ricoverato?
1 Pessimo
2 Scarso
3 Sufficiente
4 Buono
5 Ottimo

IV) Come giudica la pulizia e lôordine della stanza del reparto dove il bimbo è stato ricoverato?
1 Pessimo
2 Scarso
3 Sufficiente
4 Buono
5 Ottimo

V) Come giudica la pulizia e lôordine del reparto dove il bimbo è stato ricoverato?
1 Pessimo
2 Scarso
3 Sufficiente
4 Buono
5 Ottimo

PDF Complete
Your complimentary use period has ended. Thank you for using PDF Complete.
Click Here to upgrade to Unlimited Pages and Expanded Features

[illegible]ei pasti?
[illegible]
5 Ottimi

VII) Come giudica l'attenzione del reparto al dolore provocato nel bambino dalla malattia e/o dalle manovre assistenziali?
1 Pessima
2 Scarsa
3 Sufficiente
4 Buona
5 Ottima

VIII) Come giudica la disponibilità e la cortesia dei medici?
1 Pessima
2 Scarsa
3 Sufficiente
4 Buona
5 Ottima

IX) Come giudica la disponibilità e la cortesia degli infermieri?
1 Pessima
2 Scarsa
3 Sufficiente
4 Buona
5 Ottima

X) Come giudica le informazioni sulla malattia del bambino che ha ricevuto durante il ricovero?
1 Pessime
2 Scarse
3 Sufficienti
4 Buone
5 Ottime

Your complimentary use period has ended. Thank you for using PDF Complete.
Click Here to upgrade to Unlimited Pages and Expanded Features

ALLEGATO 3

BENESSERE DEL CANE DURANTE LE SEDUTE

Tipo di intervento e utenza:
Inizio attività ore　　　　　　　　Fine attvità ore　　　　Tempo di impiego totale
Numero utenti con cui lôanimale ha avuto contatto:

COMPORTAMENTO			DURATA (TEMPO)				
Ansimazione	no	si	< 25%	25%	50%	75%	>75%
Aumentata vigilanza	no	si	< 25%	25%	50%	75%	>75%
Aumentata attività motoria	no	si	< 25%	25%	50%	75%	>75%
Aumentata tensione muscolare	no	si	< 25%	25%	50%	75%	>75%
Incapacità di sedersi	no	si	< 25%	25%	50%	75%	>75%
incapacità di rilassarsi	no	si	< 25%	25%	50%	75%	>75%
Corrugamento della fronte	no	si	< 25%	25%	50%	75%	>75%
Richiesta di attenzioni	no	si	< 25%	25%	50%	75%	>75%
Sbadigli ripetuti	no	si	< 25%	25%	50%	75%	>75%
Leccamento delle labbra	no	si	< 25%	25%	50%	75%	>75%
Orecchie appiattite dietro alla testa	no	si	< 25%	25%	50%	75%	>75%
Tremori	no	si	< 25%	25%	50%	75%	>75%
Pupille dilatate	no	si	< 25%	25%	50%	75%	>75%
Eliminazione di urina e feci	no	si	< 25%	25%	50%	75%	>75%
Vocalizzazione	no	si	< 25%	25%	50%	75%	>75%
Postura bassa	no	si	< 25%	25%	50%	75%	>75%
Posizione bassa della coda	no	si	< 25%	25%	50%	75%	>75%
Ricerca di un posto per nascondersi	no	si	< 25%	25%	50%	75%	>75%
Tentativi di evitamento	no	si	< 25%	25%	50%	75%	>75%
Tentativi di fuga	no	si	< 25%	25%	50%	75%	>75%
Le palpebre diventano ñpesantiò	no	si	< 25%	25%	50%	75%	>75%
Freezing (inibizione)	no	si	< 25%	25%	50%	75%	>75%
Diminuita attività motoria	no	si	< 25%	25%	50%	75%	>75%
Diminuita esplorazione	no	si	< 25%	25%	50%	75%	>75%
Aggressione difensiva	no	si	< 25%	25%	50%	75%	>75%

PDF Complete
Your complimentary use period has ended. Thank you for using PDF Complete.
Click Here to upgrade to
Unlimited Pages and Expanded Features

ALLEGATO 4

Razza: Golden Retriver biondo

PERCORSO ENTRATA

1) Entrata al piano terra dalla parte del DH endocrinologico seguendo il percorso indicato dalla figura 1, fino alla scala

Figura 1

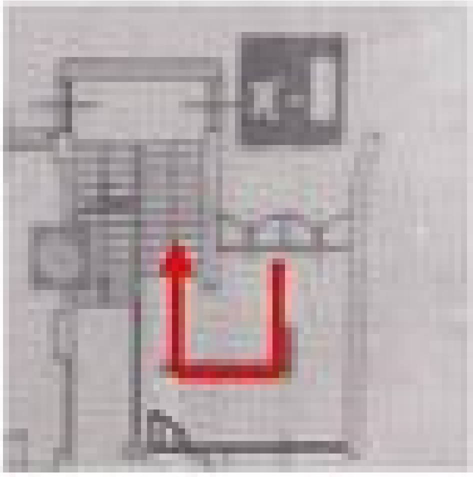

2) Accesso al secondo piano tramite scala interna

3) Entrata in reparto dalla parte della biblioteca e accesso alla stanza di degenza seguendo il percorso indicato dalla figura 2.

Figura 2

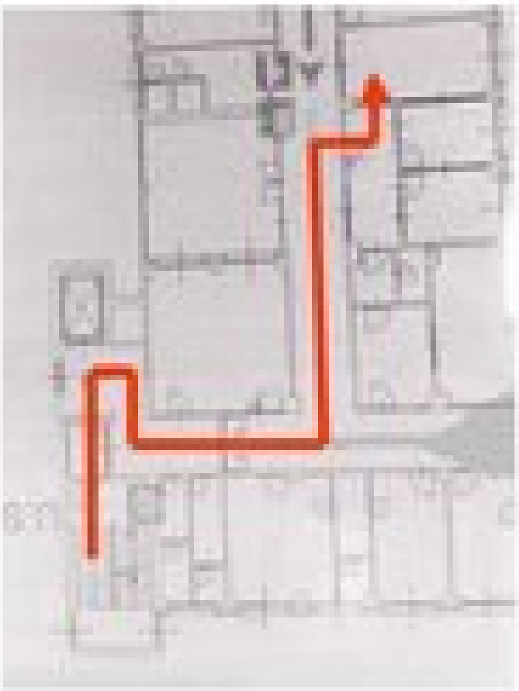

PERCORSO USCITA

Per lûscita il cane verrà accompagnato attraverso lo stesso percorso utilizzato nellêntrata
