# Supplementary material for: Post-Operative Benefits of Animal-Assisted Therapy in Pediatric Surgery: A Randomised Study
Source: PLoS One. 2015 Jun 3;10(6):e0125813. doi: 10.1371/journal.pone.0125813 (PMC4454536; doi:10.1371/journal.pone.0125813)
Supplement: S2 Protocol — (PDF) [file pone.0125813.s003.pdf]

ClinicalTrials.gov
A service of the U.S. National Institutes of Health

Animal-assisted Therapy in Pediatric Surgery: Post-operative Benefits

This study has been completed.

Sponsor:
IRCCS Policlinico S. Matteo

Information provided by (Responsible Party):
Pelizzo Gloria, IRCCS Policlinico S. Matteo

ClinicalTrials.gov Identifier:
NCT02284100

First received: October 29, 2014
Last updated: November 3, 2014
Last verified: November 2014
History of Changes

Purpose

The relationship between human beings and **animals**, especially dogs, has existed for thousands of years. Historically, **animals** have held an important role in this relationship as they provide company, stimulus and motivation. **Animals** are excellent company, since their visitation they do not discriminate or segregate any person, that is, they are free of prejudice.

In spite of the long-lasting presence of companion **animals** in human life, the idea that interaction with **animals** may exert a positive effect on human health is rather recent.

The American Veterinary Medical Association classifies therapeutic **animal assisted** interventions (AAI) into three categories: **animal assisted** activities (AAA) that utilize companion **animals**; **animal assisted therapy** (AAT) that utilizes **therapy animals** and service **animal** programs (SAP) that utilize service **animals**. AAT in particular, is a goal-directed intervention in which an **animal** that meets specific criteria is an integral part of the **treatment** process. AAT is technically defined as the use of trained **animals** by trained health professionals to facilitate specific, measurable goals for individual patients for whom there is documentation of progress .

Interest in AAT has been fueled by studies supporting the many health benefits. AAT has proven a useful adjunct in a variety of settings including mental health facilities, nursing homes and hospitals where most studies have been performed with adult patients with variable interventions, goals, patient characteristic and patient needs. In these studies, AAT resulted in significant reductions in anxiety, agitation and fear. In children, AAT dogs decreased distress during painful medical procedures, promoted calmness in children with post-traumatic stress disorders and increased attention and positive behaviors in children with pervasive developmental disorders.

Surgical procedures and hospitalization can be stressful for both children and their parents and they are associated with pain, helplessness, fear and boredom. AAT has been shown to facilitate a child's ability to cope with hospitalization, but to date, no studies on AAT benefits in pediatric surgery have been reported.

The purpose of this study was to better understand the effects of an AAT program on neurological, cardiovascular and endocrinological responses to stress and pain in the immediate post-operative period in children undergoing surgical procedures.

Condition	Intervention
Surgery	Behavioral: **animal assisted therapy**

Study Type: Interventional
Study Design: Allocation: Randomized
Intervention Model: Parallel Assignment
Masking: Open Label
Primary Purpose: Supportive Care

Official Title: **Animal-assisted Therapy** in Pediatric Surgery: Cardiovascular, Neurological and Endocrinological Responses to Stress and Pain in the Immediate Post-operative Period

Further study details as provided by IRCCS Policlinico S. Matteo:

Primary Outcome Measures:

- Neurological impact [Time Frame: Partecipants will be followed for the duration of immediate postoperative period, an expected average of 4 hours] [Designated as safety issue: No]

  Neurological impact assessed by the difference in prevalence of beta (>14 Hz) electroencephalogram (EEG) activity between intervention and

control group.

Secondary Outcome Measures:

- Autonomic impact [Time Frame: Partecipants will be followed for the duration of immediate postoperative period, an expected average of 4 hours] [Designated as safety issue: No]

  Autonomic impact assessed by the difference in blood pressure (BP) between intervention and control group

- Cardiac impact [Time Frame: Partecipants will be followed for the duration of immediate postoperative period, an expected average of 4 hours] [Designated as safety issue: No]

  Cardiac impact assessed by the difference in heart rate between intervention and control group

- Respiratory impact [Time Frame: Partecipants will be followed for the duration of immediate postoperative period, an expected average of 4 hours] [Designated as safety issue: No]

  Respiratory impact assessed by the difference in oxygen saturation (SpO2) between intervention and control group

- Cerebral oxygenation [Time Frame: Partecipants will be followed for the duration of immediate postoperative period, an expected average of 4 hours] [Designated as safety issue: No]

  Cerebral oxygenation assessed by the difference in prefrontal oxygenation (HbO2) between intervention and control group

Other Outcome Measures:

- Endocrinological impact [Time Frame: Partecipants will be followed for the duration of immediate postoperative period, an expected average of 4 hours] [Designated as safety issue: No]

  Endocrinological impact assessed by the difference in salivary cortisol levels between intervention and control group.

Enrollment:	40
Study Start Date:	September 2013
Study Completion Date:	September 2014
Primary Completion Date:	April 2014 (Final data collection date for primary outcome measure)

Arms	Assigned Interventions
Experimental: **animal assisted therapy** group<br>the dog was present during post-operative awakening (2 hours after surgery)	Behavioral: **animal assisted therapy**<br>For the AAT session, a 7 year old Golden Retriever was employed as the **therapy animal**. Prior to the study, the dog underwent rigorous screening although she had previous experience in **Animal Assisted** Interventions and was already trained and prepared prior for this type of work. The dog was fully vaccinated, bathed regularly, screened for enteric pathogens, and treated for internal and external parasites on a monthly basis. The dog and handler met hospital policy for participating in **animal-assisted therapy**, including documentation of the dog's current vaccinations, controllability and temperament.<br>The welfare of the dog was monitored and certificated by a dedicated veterinary during and at the end of the all sessions.
No Intervention: standard group<br>children had standard post-operative medical care	

Show Detailed Description

Eligibility

Ages Eligible for Study:	3 Years to 17 Years
Genders Eligible for Study:	Both

Accepts Healthy Volunteers: No

Criteria

Inclusion Criteria:

- immunocompetent children (both genders)
- aged 3 to 17 years
- undergoing surgical procedures (including orchidopexy, inguinal or umbelical hernia repair, circumcision, varicocele treatment)

Exclusion Criteria:

- allergy or fear of dogs
- previous AAT experience
- immunodeficiency
- chronic illness
- obesity and use of any medications.

Contacts and Locations

Choosing to participate in a study is an important personal decision. Talk with your doctor and family members or friends about deciding to join a study. To learn more about this study, you or your doctor may contact the study research staff using the Contacts provided below. For general information, see Learn About Clinical Studies.

No Contacts or Locations Provided

More Information

No publications provided

Responsible Party:	Pelizzo Gloria, Professor, IRCCS Policlinico S. Matteo
ClinicalTrials.gov Identifier:	NCT02284100 History of Changes
Other Study ID Numbers:	20130005132
Study First Received:	October 29, 2014
Last Updated:	November 3, 2014
Health Authority:	Italy: Ethics Committee

ClinicalTrials.gov processed this record on November 04, 2014
